# Biometric authentication systems: Trends, challenges, and future prospects – A comprehensive review

**DOI:** 10.1016/j.mex.2026.103908

**Published:** 2026-04-09

**Authors:** Shital Kakade, Umesh Raut

**Affiliations:** Department of Computer Engineering and Technology, Dr. Vishwanath Karad MIT World Peace University, Kothrud, Pune, Maharashtra 411038, India

**Keywords:** Unimodal, Multimodal, Biometric, Preprocessing

## Abstract

With cyber security concerns high on the list of issues, it is no surprise that demand for secure confidentiality for all users and objects is at an all-time high; at the same time, the development of robust reliable systems for providing confidentiality is also at an all-time high due to the increasing degree of use of the internet. Systems will utilize the authenticated user identifying that combines both provided user identifying information with data stored in a secure database. Traditional authentication methods (passwords and pin numbers) have been proven inadequate to provide robust security and improve user experience with the rise of cyber security threats. Individual unimodal biometric systems face multiple challenges. For this reason, a multi biometric authenticating system (using more than one biometric characteristic) would be a viable solution to the limitations of unimodal biometric authentication systems by providing greater robustness and reliability to the authentication process. Multimodal biometric authentication systems leverage several biometric traits, to boost the security and accuracy of identity verification. The proposed system has four major components such as preprocessing, feature extraction, feature fusion, and authentication. By merging data from multiple modalities, these systems get around the limitations of unimodal biometric systems.

## Review Highlights

• More secure compared to single-modal biometrics.
• Harder to falsify multiple biometric traits simultaneously.
• Reduces dependency on a single biometric, allowing alternative options for users.

## Graphical abstract

**Figure:**
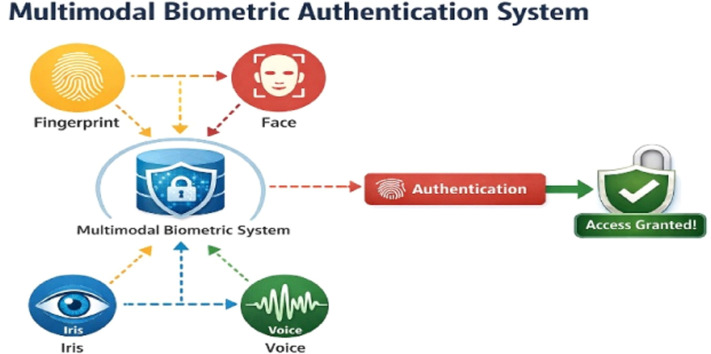

## Specifications table

**Table:** 

**Subject area**	Computer Science
**More specific subject area**	Deep Learning
**Name of the reviewed methodology**	Survey research papers
**Keywords**	Unimodal, Multimodal, Biometric, preprocessing
**Resource availability**	Not applicable
**Review question**	Q1. Does the paper clearly explain why multimodal biometric authentication is needed compared to unimodal systems? Q2. Does the introduction properly explain the importance of multimodal biometrics in modern security systems? Q3. Are the major biometric modalities discussed adequately?

## **Background**

Authentication is the technique or process to identify the authorized users in order to use stored data in a matching process to give system access control [1]. Now a days people have been paying additional attention to information security as a direct outcome of advances in science and technology [4,5]. Conventional authentication approaches, such as using passwords with account numbers are simply substituted by pretending to be someone else once they are stolen [4]. One of the utmost promising security technologies of this century is biometrics, which is rapidly developing. To identify a person, it uses a number of techniques, including fingerprints, iris, face, hand geometry, voice, signature, keyboard typing pattern, etc. [7,8]. Environmental conditions can generally cause biometric data to be corrupted or distorted. Biometric traits can be divided into two categories: intrinsic and extrinsic [5]. Extrinsic Biometric traits are observable and subject to outside influences, but intrinsic Biometric traits are unaffected by outside influences [6,8]. Additionally, there are two categories of Biometric systems: unibiometric authentication systems and multi-biometric authentication systems [39]. For user authentication, a unibiometric system depend on a single biometric feature, such as either voice or facial biometric feature [1,6]. However, for the multi-biometric system to work, multiple biometric features need to be integrated into a single authentication decision [1,6]. Furthermore, a broader spectrum of individuals, including amputees and those with physical disabilities, who are unable to produce traditional biometrics like fingerprints, palm prints, or iris, can provide an ECG. Additionally, ECG data can be collected from several portions of the human body such as the fingertip, which allows it to be used by the greatest number of people [1,12,41,42].

For recognition of any object, most of the biometrics researchers rely on machine learning techniques. Prior to categorizing unprocessed biometric data, machine learning (ML) algorithms [14] must modify the data into an appropriate format and extract features from the data. Moreover, ML algorithms need some pre-processing phases to be carried out preceding to feature extraction [14,18]. Additionally, certain extraction techniques don't always function well with several biometric forms or data sets of identical biometrics [18]. Also, this mechanism cannot survive with biometric image transformations, for example, rotation and zooming [15].

Recently, deep learning has made a substantial impact and produced outstanding outcomes in biometrics systems. The DL algorithm has overcome various restrictions of supplementary machine learning algorithms, mostly those accompanying with feature extraction techniques [6,16,24,26,28]. Also, the DL algorithms are capable of extracting features from raw data and handling biometric image modifications [[6], [26], [34]]. Moreover, CNN was only model deployed by many researchers for biometric authentication. However, CNN does not guarantee obtaining optimum results [27]. Also, other ensemble DL techniques has quite more limitations such as over-fitting issues, vulnerable authentication, high error rates, computational complexity, and less security [19,15].

The core contributions of this paper are as follows:

• **Comprehensive Coverage:** This review consolidates recent research across multiple biometric traits, including fingerprint, finger vein, face, iris, and voice recognition.
• **Focus on Deep Learning:** It provides an in-depth analysis of deep learning-based approaches, highlighting their advantages, limitations, and practical deployment challenges.
• **Multimodal Biometrics Analysis:** The paper examines various multimodal fusion strategies and compares their effectiveness in improving recognition performance and robustness.
• **Comparative Evaluation:** The study includes detailed comparative tables summarizing methods, datasets, performance metrics, and limitations.

### Literature search methodology

This search method was both systematic and reproducible, collecting literature concerning biometric authentication systems. This represents a systematic and methodologically sound approach to ensure rigor, transparency and breadth of existing knowledge.

**Step 1: Selection of Data Sources**

The literature search was conducted with several well-known academic databases, including Scopus, Web of Science, IEEE Xplore, ScienceDirect, SpringerLink and Google Scholar. The selection of these sources was based on their established importance and reliability in indexing peer-review material within computer science, engineering, and the biometric security domain.

**Step 2: Search Strategy and Query Formulation**

A structured search strategy was created with relevant keywords combined with Boolean operators to enhance the specificity of the results. The keywords covered for searching were “biometric authentication”, “multimodal biometrics”, “face recognition”, “finger vein recognition”, “ECG-based authentication” and “deep learning in biometrics”. Boolean operations: AND, OR [6] were used to further refine the search. We utilized search queries such as “biometric authentication AND deep learning” and “multimodal biometrics AND (ECG OR finger vein)” to extract the studies that are most relevant to this review.

**Step 3: Time Frame Definition**

The search was time-bound and limited to studies published within the range of 2015 until 2025.

**Step 4: Inclusion and Exclusion Criteria**

To control for superiority and significance of selected studies, pre-defined inclusion and exclusion criteria were used.

Inclusion criteria were peer-reviewed papers and conference works published in English that proposed biometric authentication methods (including both unimodal and multimodal systems), datasets, performance metrics, and security challenges.

Exclusion criteria required to eliminate duplicates, studies not directly related to biometric authentication, non-peer-reviewed content (e.g., editorials, blogs), and articles with insufficient methodological or experimental details.

**Step 5: Study Selection Process**

Study selection was done in several phases. Initially over 150 papers were finally identified using databases. After deletion of duplicate studies, the titles and abstracts were screened for relevance. We conducted a full-text review of the shortlisted articles to assess their relevance according to the criteria set out at the beginning. Eventually, a total of 50 articles were chosen to analyze.

**Step 6: Data Extraction and Synthesis**

Data extraction of the key information was done from selected studies in a systemic way such as biometric modalities, techniques used, datasets, performance metrics (increase and importance), applications along with strength and weaknesses. The data collected were processed to be able to summarize it in an organized manner, identify trends and gaps while identifying commonalities between biometric authentication systems.

## Method details

### Necessity of biometric systems

Biometric systems are becoming more and more essential [7] in various sectors due to the growing demand for security, accessibility, and effectiveness. Here's a breakdown of the key reasons why biometric system is necessary: -

1. **Enhanced Security-**
  Unique Identification: Biometrics provide a safer method of identification [30] than conventional password-based systems, as each individual's biometric traits (like fingerprints, finger vein, facial expressions [38], voice, or iris patterns) are unique. This makes it considerably harder to mimic someone.
2. **Difficult to Forge**:
  The manner in which biometric authentication differs from traditional authentications such as passwords and PINs is the fact that biometric information is considerably more difficult to copy or steal, thereby providing users with an added barrier of security [39].
3. **Convenience and User Experience:**
  Biometric authentication is convenient and enhances the user's experience because it does not require users to remember and enter complicated passwords, nor does it require users to physically carry security tokens. Because of this, the authentication process is much quicker for a user: by simply scanning their fingerprint, taking a picture of their face, or scanning their retinas.
4. **Contactless Options**:
  With Biometric Authentication options available in a Contactless manner, a user will have access to methods of authentication that are not reliant on physical contact with the authentication device. The main ways that Biometric Authentication is used in a Contactless manner include, but are not restricted to, Facial Recognition and Iris Scanning.
5. **Fraud Prevention**
  A major benefit of using biometrics for authentication is that it can significantly reduce the risk of fraud. Because biometric systems only allow access to the owner or authorized users, it is very difficult for someone else to use your biometric data to commit fraud by impersonating you or using your information. As such, there is less chance that someone will impersonate you through your biometric information than through traditional identification methods. Furthermore, the combination of biometric authentication with other forms of authentication (such as passwords, PINs) creates a stronger layer of security than either method can provide on their own.
6. **Cost Savings:**
  Organizations implementing biometric authentication systems have reduced losses resulting from unauthorized access and fraud, as the improved security provided by these systems has led to decreased losses. Most often, the savings created by providing a secure means of authentication will be greater than the initial expenses involved in implementing biometric authentication systems. Lower Operational Costs: Biometric systems can reduce the need for physical security measures, personnel, and manual checks, leading to cost savings in long-term operations.

### Classification of biometric systems

A. Unimodal Biometric System
B. Multimodal Biometric System

**A. Unimodal Biometric System**-

A unimodal biometric system relies on a individual biometric trait, such as fingerprint, face, iris, voice, or electrocardiogram (ECG), for user authentication or identification [30]. In this case of authentication, the captured biometric sample is compared against a collection template which has been stored, allowing you access if enough match is available. Such systems are usually adopted because of their ease, low cost and easy implementation. However, they face a number of limitations such as noise in sensed data, intra-class variance, and inter-class similarity which can lead to accuracy loss. Moreover, other factors contributing to their unreliability include non-universality and susceptibility towards spoofing attacks. Overall, while unimodal systems are efficient and practical, their inherent limitations restrict their effectiveness in high-security applications. This system may face with following problems-

1. **Vulnerability to False Rejection or Acceptance**: If the biometric data is of poor quality (e.g., due to dirt on a fingerprint scanner, poor lighting for facial recognition), the system may fail to correctly authenticate the user. This can lead to a high rate of false rejections or false acceptances.
2. **Single Point of Failure**: If an individual’s biometric trait is compromised (e.g., fingerprints are stolen or a facial image is spoofed), the entire system can be bypassed or compromised.
3. **Limited Accuracy and Robustness**: Age, injury, environmental factors, and changes in the biometric trait over time can all have an impact on how accurate unimodal systems are.

**B. Multimodal Biometric System**-

Multimodal biometric system uses multiple i.e. more than one biometric trait or modalities to authenticate or identify individuals [7]. Instead of relying on a single biometric trait, it integrates biometric information from several sources, such as fingerprint [20,40,28] facial features [3,6,10,20], iris [6,9,16] voice [27,32], ECG [2,14] and even behavioural patterns, in order to enhance the accuracy, security, and dependability of biometric authentication. Although traditional biometric technology has limitations of false negatives and false positives, multimodal provides a way to reduce errors by combining multiple forms of biometrics together. Combining multiple pieces of biometric data reduces the likelihood of false negatives or positives, improving accuracy, security and reliability in comparison to traditional methods of checking one form of biometric identification alone. Another benefit to combining multiple pieces of biometric data together within a single system is the ability for that same system to easily adapt to user needs at any point in time and to allow for biometric authentication in a variety of different environments. Thus, multimodal biometric systems can serve a greater purpose in a manner requiring constant verification of high security applications. In multimodal biometric system the modalities might be combined in various ways, such as:

1. **Serial Fusion**: Biometric traits are captured and processed sequentially (one modality after another).
2. **Parallel Fusion**: Several biometric traits are captured at the same period, and the system processes them simultaneously.
3. **Hybrid Fusion**: A combination of both serial and parallel fusion techniques.

The system can compensate for the limitations or restrictions of a single biometric modality by relying on others. For example, if a fingerprint scan fails due to dirt or injury, the face or voice recognition can still verify the identity. Combining multiple modalities increases the security of the system because the likelihood of both modalities being compromised at the same time is much lower. For instance, it would be much harder to spoof both a voice and a fingerprint compared to just one modality. Multimodal systems can be more user-friendly because they are less likely to reject users who might experience issues with one biometric trait (e.g., a fingerprint scanner not working due to a cut or dirt on the finger). As a result, users have a more satisfying experience and will not get as frustrated during their authentication process. Because it would be extremely difficult for fraudsters to impersonate or steal multiple different biometric characteristics at the same time, the system is much more difficult to trick as opposed to a unimodal system, which is more vulnerable to certain types of attacks (e.g., counterfeit fingerprints or facial recognition). (Table 1)

**Table 1:** Comparison of unimodal and multimodal biometrics.

Parameter	Unimodal Biometric Systems	Multimodal Biometric Systems
**Definition**	Uses a single biometric trait	Uses multiple biometric traits
**Accuracy**	Moderate	High
**Security**	Lower; vulnerable to spoofing	Higher; resistant to spoofing
**Robustness**	Sensitive to noise and variations	More robust due to data fusion
**Universality**	Limited (may not suit all users)	Improved (alternative traits available)
**Complexity**	Low	High
**Cost**	Low	High

### Types of biometric systems

Biometrics states to the study or analysis of distinct features of a person's body and behavior for either identifying or verifying that person [2]. The following summary provides further details about the types of biometrics (i.e., both physical and behavioral characteristics). Physical biometric measures analyze and classify unique physical characteristics of the individual that are part of his/her body, while behavioral biometrics measure and classify unique characteristics associated with the individual's behavior. Physical biometric measurements typically demonstrate stability over time, thus providing a reliable source of information to verify someone's identify or to authenticate someone's identity. Behavioral biometrics analyze an individual’s distinct patterns of behavior to identify or authenticate them. Unlike physical biometrics, which rely on physical traits, behavioral biometrics concentrate on in what way actions are performed [2] often integrating continuous monitoring to ensure ongoing security. Following are the some commonly used biometric systems –

• **Face:** Face biometrics refers to the technology and methods used to recognize or verify an individual by considering their facial characteristics [3]. It is a subset of biometric systems, which employ physical or behavioural traits to authenticate identity. It captures and analyses facial landmarks (e.g., distance between eyes, nose shape) using cameras and algorithms like deep learning. It is commonly used for security, authentication, and surveillance purposes. In facial recognition system detects and analyses facial features and compares these features to stored data for identification or verification [38]. For a facial recognition system to work effectively in real life, it must automatically capture an image or video of the face using a camera. Preprocessing enhances the image for better feature recognition [6,10,20]. During feature extraction analyses distinctive facial features like the jawline, nose shape and distance between eyes using specific algorithms. Compares the features that were extracted with the database's previously stored templates [17].
• **Fingerprint:** Fingerprint biometric authentication is a widely used technology that verifies a person's distinctiveness by analysing unique patterns and ridges on their fingerprints [20]. It is a secure, reliable, and convenient method for identity verification and access control. A fingerprint scanner is used to captures the fingerprint image using optical, capacitive, or ultrasonic sensors. Extracted fingerprint features are converted into a digital template and securely stored in a database or device. During authentication, a new fingerprint is scanned and compared with stored templates to confirm identity [40].
• **Iris:** The technique for identifying an individual's identity based on the unique characteristics found in the pattern of their iris is referred to as iris biometric authentication. The iris is the coloured area of the eye that appears circular around the pupil, and the vast array and complexity of the patterns are unique to every individual and remain unchanged during their lifetime, hence making it such a valuable biometric identifier [6]. A digital camera specifically designed for taking high-resolution images of the iris captures a photograph in an infrared wavelength, allowing for greater detail and less reflection. Once the iris photograph has been captured, the iris will be extracted from the rest of the photograph by removing portions of the photograph that include the eyelid and eyelash as well as reflections that were captured when the photograph was taken. Unique features, such as ridges, furrows, and patterns within the iris, are extracted and encoded into a digital template. The iris template is securely stored in a database or on the user's device. A completely new image of the iris is taken during authentication, and it is compared with the template that has been saved. They are allowed access if they match [9,16].
• **Voice:** Voice biometric authentication is a technology that securely and conveniently verifies a person's identity using their distinctive voiceprint [27]. It analyses both the physiological (vocal tract shape) and behavioural (speech patterns) traits of a person's voice to create a distinct voiceprint for authentication. It depends on the distinct qualities of an individual's voice, including pitch, tone, cadence and speech patterns, which are difficult to replicate. The user speaks a predetermined phrase or sentence into a microphone or phone [32]. The spoken input can be text-dependent (fixed phrase) or text-independent (any phrase). The system analyses unique voice characteristics such as pitch, tone, cadence, and accent. The process of analysing the user's voice is performed using Spectral Features (Formants, Pitch Contours, and Energy Distribution) to extract spectral characteristics from the user's speech to form a digital voiceprint that can then be securely saved in the biometric identification system. The digital voiceprint is then compared against the user's voice during authentication to authenticate their identity.
• **Hand geometry:** Hand geometry is a method of biometric authentication in which a person's unique physical characteristics of their hand are used to verify their identity. The measurement of the hand is based on several factors, including the length, width, thickness, and shape of the fingers and palm. This method is quick to perform and is quite accurate; therefore, many applications requiring moderate security have used this method. To use hand geometry biometrics, the user must place their hand on a flat surface or scanner to ensure that the placement is correct. This is accomplished by the use of guiding pins or something similar. After positioning, a photograph is taken of the user's hand with either a 2D or 3D camera to capture all of the information needed to determine the user's hand geometry. Once this information has been collected, the biometric system stores a unique hand geometry template that will be used later for user authentication. Once the user wants to access the system, they may again put their hand in the correct position for a second digital image to be captured. The measurement from the current photograph will be compared against the stored hand geometry templates for a match, verifying the user's identity.
• **Keystroke:** By examining the unique typing habits of an individual, keystroke biometric authentication uses the way each person types to verify their identity [33]. In addition to examining the speed, consistency, and other aspects of typing with a computer keyboard or keypad, several other factors are taken into consideration during the process of determining the identity of a user through this method. Typing information is obtained whenever a user uses a keyboard, whether by entering passwords, typing certain phrases, or general typing activities. Certain Key features are extracted from the user's keystroke behavior [33]. Once these features are extracted, they are assembled into a specific template for that particular user. When a user attempts to authenticate using a currently typed example, this typing example is checked against the previously stored user template. If the two templates match with an acceptable level of similarity, the user will be authenticated and allowed access.
• **ECG:** Using the electrical signals of the heart for identification, or electrocardiogram (ECG) biometric identification, as a physiological form of biometric security [1,12]. By correlating an individual's unique heartbeats with an ECG device provides for secure identification. This is accomplished by placing electrodes on the body (e.g., fingertips, wrist or chest) to receive the heart's electrical signals. ECG signals are also recorded by wearable devices (e.g., smartwatches, fitness bands) [12]. The analysis of the ECG waveform allows for identification and retrieval of the unique features that are then used to generate a unique ECG biometric template. When an individual attempts to gain access, the system collects a live signal from an ECG and compares it with the stored template [41,42]. Access is granted if the live ECG matches the template according to pre-defined criteria.
• **Finger vein:** Finger vein biometric authentication is a secure and progressive method that verifies a individual's identity by analysing the unique pattern of veins inside their finger [1,13]. It is a contactless or minimally invasive technique that relies on near-infrared (NIR) light to capture vein patterns, which are very difficult or impossible to reproduce or forge. To capture the image user places their finger on or near a scanner, NIR light passes through the finger, and the haemoglobin in the blood absorbs the light, making the veins visible [6,24]. A high-contrast image of the vein pattern is taken from the system. The vein pattern contains unique points of interest including branching points, crossings, and the width of the vein. These characteristics are used to create a digital copy of the vein pattern that is then stored securely. When the individual comes to be authenticated, their current vein pattern will be scanned and compared to the stored version [28,31]. If the vein patterns are the same then the individual will have access.

The following is an evaluation of different biometric methods according to measures like applications, cost, accuracy, and usability: (Table 2)

**Table 2:** Comparison of different biometric features.

Biometric Feature	Uniqueness	Accuracy	User Acceptance	Cost	Key Limitation
**Fingerprint**	High	High	High	Low	Affected by cuts, dirt, wear
**Face**	Moderate	Moderate	High	Low	Sensitive to lighting, pose
**Iris**	Very High	Very High	Moderate	High	Requires specialized sensors
**Voice**	Moderate	Moderate	High	Low	Affected by noise, illness
**ECG**	High	High	Moderate	Moderate	Requires physical contact/sensors
**Finger Vein**	High	High	Moderate	High	Expensive acquisition device

### Fusion techniques in multimodal biometrics

Fusion methods of combining information from multiple biometric modalities offer the potential to enhance the performance and reliability of biometric systems. Fusion methods can occur at multiple levels of a biometric system:

1. **Sensor level fusion:** Combines the raw outputs of multiple biometric sensors, such as fingerprints, facial images, or voice recordings, prior to the feature extraction step in order to maximize the quality of the incoming signals and reduce background noise; however, this method requires that the sensors be highly compatible. In sensor level fusion, the incoming outputs of all the sensors are combined to create combined raw output [1,6].
2. **Feature level fusion:** Combines all of the individual features extracted from the biometric inputs of all sensors to create one single binary feature vector, thus forming a composite feature vector of biometric information from the biometrics on all of the biometric input sensors. By merging individual feature sets in this way, a comprehensive feature representation is generated allowing for improved classification accuracy. While feature level fusion provides high discrimination capabilities, it requires very sophisticated methods for feature normalization and increased storage [1,3,6,16,27,37,37]
3. **Score-Level Fusion:** The goal of Score-Level Fusion is to combine the individual scores generated by all of the biometric systems (modalities). The most common methods for this type of fusion are: Sum Rule, Weighted Sum, and Min-Max Normalization. Score-Level Fusion makes it possible to combine the contributions from each of the systems in such a way that the best performance can be achieved from the different types of biometric data being used. Once each modality has been processed, and individual biometric samples matched to the appropriate system, the final decision to verify or reject the candidate will be based on the combined score. Score-Level Fusion can be done with weighting to discriminate among the different types of systems, giving more weight to the systems that have higher accuracy, or based on user preferences [20,22,24,37,37].
4. **Decision-Level Fusion:** The goal of Decision-Level Fusion is to combine the individual decision outputs generated by each of the biometric systems (modalities) into a single definitive decision concerning the identification of the individual. While this type of fusion is easier to implement than either Feature or Score-Level Fusion, it can also produce very good results with respect to matching candidates. Decision-Level Fusion can be done using a variety of methods, such as Majority Voting or using "AND/OR" rules to combine the individual decisions made by the multiple biometric systems. Decision-Level Fusion can be advantageous when it is impractical to use either Feature or Score-Level Fusion. One drawback of Decision-Level Fusion is that some information is lost compared to lower class fusion methods [22,25]. (Table 3)

**Table 3:** Multimodal fusion techniques.

Fusion Level	Description	Example	Advantages	Limitations
Sensor Level	Combine raw data	Face + IR image	Rich information	Complex preprocessing
Feature Level	Combine features	Face + Fingerprint features	High accuracy	High dimensionality
Score Level	Combine scores	Weighted sum	Easy to implement	Less optimal
Decision Level	Combine outputs	Majority voting	Simple	Lower performance

All of these fusion levels should be used only for their intended purposes based on the specific requirements of a particular biometric system as well as the computational capabilities present within that system.

### Related work

In the past several years, many researchers have produced various frameworks for a variety of multimodal biometric authentication systems.

As an example of this emerging research, El-Rahiem et al. [1] developed a novel multimodal biometric authentication system that uses Deep learning techniques for combining electrocardiogram (ECG) data with finger vein data. This combination enhances security and reliability through a hybrid model that incorporates ECG, which generates a specific physiological signature from the heart's activity, together with finger veins' anti-counterfeit capabilities which helps to resolve issues encountered by faculty-authentication based systems. El-Rahiem et al. used a 'Deep Fusion' framework for combining data from both modalities throughout the various points within the Processing Network; furthermore, they utilized Convolutional Neural Networks (CNN) for generating highly accurate feature extraction, ultimately leading to significantly increased accuracy of decision-making as a result of the fusion technique employed.

The study results indicated that the Deep Fusion of finger veins with ECG resulted in performance levels significantly greater than those of unimodal authentication approaches [1]. More precisely, the authors reported that findings showed that proposed methods offered improved accuracy for recognizing biometric properties while demonstrating decreased vulnerability to environmental changes. The authors maintain that their methodology for implementing biometric authentication can effectively solve the challenges associated with deploying biometric authentication techniques in the real world and, therefore, can provide an attractive solution for use in highly regarded high-security environments requiring a very high degree of assurance of authenticating users and providing protection from fraudulent impersonation attempts.

The edited volume, Hidden Biometrics: Where Biometric Security Meets Biomedical Engineering [2], N. A. Amine (author and editor), details the convergence of Biometric Security and Biomedical Engineering and how they are both being developed. The central focus of this edited volume is the use of hidden biometric identifiers for security; this is to say that hidden biometric identifiers are not taken from people (like their finger or face) but come from physiological signals (such as Electrocardiogram (ECG), Electroencephalogram (EEG)), among other things. The hidden biometric identifiers that will be described are obtained from the same physiological signals that are used to obtain the more familiar (external) biometric identifiers; however, because of their similarity to the other physiological signals, hidden biometric identifiers are less easily counterfeited or tampered with than external biometric identifiers (like fingerprints and facial recognition).

In order to overcome the above limitations, Wang et al. proposed a Novel Hybrid Multimodal Biometric Recognition System using a CNN-based Model [3]. They have proposed a new way to merge the Fingerprint and Facial Biometrics (i.e., the Biometric Data from Fingerprints and Facial Features) The combination of Fingerprint and Facial Features using the Fusion Method improves the Precision with which the System Identifies Individuals. Experimental Results indicate that the CNN-based Fusion of Fingerprint and Facial Features outperformed both Unimodal Biometric System (such as a Fingerprint System or a Face Recognition System) and the Flat Biometric System with respect to Security and Recognition Performance in situations when an Individual Biometric would not have functioned.

According to Alrawili et al. [4], user authentication using biometrics has been studied through a variety of methods. This study includes both physiological (e.g., fingerprint, face, iris and vein recognition) and behavioural (e.g., voice, gait and signature) methods. They considered the key performance indicators of biometric traits, including security, usability, robustness and scalability, while also evaluating each modality based on standard metrics (e.g., FAR, FRR). The study also evaluated the appropriateness of various biometric modalities for use in static, remote, and continuous user authentication systems. The authors identify major cybersecurity threats to biometric systems, such as deepfake and replay attacks, while also identifying the many factors that may impact system accuracy (e.g., environmental variation, user variation, etc.). This survey found that unimodal systems have limitations when it comes to providing maximum levels of accuracy; therefore, multimodal systems may be more effective than unimodal systems in providing secure user authentication. Additionally, the authors provided recommendations for future research efforts to investigate the application of AI technologies and privacy-preserving biometric systems.

S.M. Abdullahi et al., in their paper on biometric template attacks and recent protection mechanisms review the different vulnerabilities of biometric authentication to several types of attacks targeting the template (i.e., the template could be stolen or altered). The authors decompose these attacks into categories such as: inversion, false-accept, hill climbing, and via record multiplicity or ARM (attacks through obtaining multiple copies of biometric information) based on what attackers know and the components of the systems. The paper reviews several of the most current biometric protection schemes (i.e., cancelable biometrics and bio-cryptosystems), and rates each based on specific criteria, e.g., Equal Error Rate (EER). In addition, the authors discuss the security/privacy/performance trade-off that exists with all security/privacy related solutions; there have been many advancements in this area, but challenges remain, especially with respect to obtaining the goals of non-invertibility and non-likability, demonstrating the need for additional, more effective, holistic frameworks for biometric template protection to be developed.

Alay et al. [6] used a deep learning-based hybrid multimodal biometric recognition system that combined the three different modalities (face, iris and vein) to identify individuals. Their paper outlines the development of that system, and describes how it works with other deep learning-based biometric methods developed by Wang et al. [3]. The authors’ hybrid system was also built as an integrated system allowing for the use of each modality's features in order to provide more secure and reliable identification and to enhance the robustness of each modality when used together in a multi-modal biometric identification system. They report that the experimental results obtained from using their hybrid system were superior to those for unimodal biometric identification systems in terms of accuracy and resistance to spoofing. Additionally, the authors conclude that the use of their fusion-based method provides higher accuracy than using any of the three modalities individually and can therefore be utilized in high security situations requiring highly accurate identification.

In their research, El-Sofany et al. [9] introduced a hybrid biometric authentication system, utilizing iris recognition to improve the security of cloud computing systems. The iris biometric modality is complemented by other authentication modalities to secure cloud computing against the vulnerabilities of existing cloud service provider (CSP) security mechanisms. The high uniqueness and stability of an individual's iris biometrics makes this method of authentication very effective. The authors feel that the primary goal of their functionally comprehensive model is to significantly strengthen the verification of a user attempting to access their data on a cloud computing system and maintain the confidentiality and integrity of stored data. The authors' performance evaluations show developments in user authentication accuracy levels and decreases in risk associated with the use of traditional computer-based user authentication methods. The authors state that this hybrid biometric and cryptographic authentication approach effectively stops unauthorized access to cloud computing systems, and they further recommend the use of this approach for all secure cloud computing applications.

K. Alkanan et al. [10] developed a multi-modal biometric authentication system as an advanced framework for security in Internet of Things (IoT) enabled smart homes by combining deep learning based facial recognition with gait based analysis. The intent of this study was to overcome the disadvantages of using single mode system that are very prone to variations in the environment such as changes in lighting, obstructed views and differences due to individual behavior. To extract face features a type of deep learning structure known as convolutional neural networks (CNNs) were used for the face recognition and for the gait analysis gait energy images (GEIs) were used to identify behavioural patterns. A weighted fusion approach was used to combine modalities to improve the robustness of the system while in dynamic conditions. The research outlined by the authors provided evidence of the effectiveness of multi-modal biometric systems by demonstrating greater accuracy (up to 92%) compared to either the face recognition/GEI alone or use of a single biometric mode (face recognition or GEI). The study provided evidence supporting the use of multimodal biometric systems to improve the security, reliability and real-time applicability of biometric systems in IoT environments.

In their recent research, A. Mansour et al. [11] proposed a Lightweight Seamless Unimodal Biometrics Authentication System with an emphasis on improving user experience and reducing the time spent authenticating when using two-factor authentication (2FA). The work introduced a new method of using an Autonomous Unimodal Biometric Authentication System (AUBAS) that uses discrete-time Markov chains as a basis for user authentication autonomously selecting the best biometric type for the user without needing any input from the user at all. There are two decision methods—likelihood-based and weighting-based—that are used to determine how AUBAS can be used effectively to authenticate users. Additionally, historical user behaviors are used to allow the system to adapt to the user as they continuously operate in a seamless manner. The results produced by simulations showed AUBAS is a substantial improvement to the user experience while still providing reliable authentication. With the capabilities offered by AUBAS, this structure will be effective for use in resource-limited scenarios such as cloud and mobile computing because it provides a viable alternative to currently used, more traditional frameworks for unimodal biometric authentication systems that consume far more resources than do AUBAS.

The framework for biometric verification through activity-aware electrocardiograms (ECGs) using deep learning on wearable devices was developed in a study by Bıçakcı Yeşilkaya and Guest [12]. The authors assessed the effect of physical activities on ECG signals and created a process that was based on activity classification prior to verifying biometrics. Multiple CNNs (Convolutional Neural Networks) were used, including ResNet50, DenseNet201, and GoogleNet, along with time-frequency representations (i.e., spectrograms and scalograms) as input data to each of the CNNs for training and testing purposes. The authors noted a lower accuracy rate in activity classification; however, the use of manual-labelled activity data increased the verification accuracy rate. The final model demonstrated to be competitive in relation to wearable devices and medical-grade devices and represented a practical solution to use for verifying biometrics in an application to establish usability in daily life. Thus, this paper has implications for how the use of biometric verification systems can be integrated into our society.

According to Zheng Hui Goh et al. [30], a multimodal biometric authentication framework (with incorporated template protection mechanisms) — which combines multiple biometric modalities (e.g., face, fingerprint, and iris) — increases accuracy and robustness of authentication processes, as well as improves resilience against spoofing attacks. One of the authors’ most significant contributions to the literature stresses the importance of protecting templates by either encrypting or transforming biometric data in order to restrict access by unauthorized users and/or to prevent reverse engineering, which thus protects the user’s privacy and prevents loss to the integrity of the system. The authors describe the overall architecture of the system, detailing key stages of the authentication process, including data acquisition, feature extraction, matching, secure storage of templates, and decision-making and demonstrate how these stages will successfully interact with each other in order to assure reliable and secure authentication results for all users of the system’s services. Furthermore, the authors evaluate performance results associated with their proposed framework using standard statistical measures such as False Acceptance Rate (FAR), False Rejection Rate (FRR), authentication accuracy, and processing overhead and conclude that their framework is a reliable and secure solution for real-world applications requiring biometric authentication.

Huang and Ma's paper presents a new method of multimodal fingerprint identification which utilizes both an asymmetric network model and a method of similarity measures [31]. Fingerprint authentication is going to be pivotal to any biometric system since it is a unique and simple method of establishing one's identity. The planned submission will present the significant role of fingerprint authentication in application areas such as access control (i.e., door locks, etc.) and forensic science. Furthermore, it will explain some of the principal challenges encountered in traditional systems of fingerprint authentication such as noise interference, variations in image capture conditions, and intra-class variability in fingerprint patterns (i.e., two fingerprints from one person). The design of the asymmetric network is likely to have two or more different branches, each providing a different manner of input data pertaining to the various modalities of the fingers, i.e., fingerprint images, vein image patterns, and geometry of a finger.

A new multimodal biometric authentication system created by Xinman Zhang [32] integrates both facial recognition and voice recognition to authenticate users of Android-based mobile devices. By using both of these modalities to authenticate, the authentication system is both more accurate and more resistant against spoofing attacks. For the purpose of creating a single unified representation of an individual that can be used to authenticate the individual, the framework incorporates modality-specific feature extraction techniques with deep learning methods being used to extract facial features, while techniques such as dynamic time warping (DTW) and convolutional neural networks (CNN) have been implemented to capture speech patterns. In addition, appropriate fusion strategies have been used to combine these features together such as feature-level, score-level, or decision-level fusion. The goal of this system is to provide the ability to deploy the authentication system on mobile devices in an efficient manner through the use of an optimized software architecture and user interface design. Furthermore, an improved local binary pattern (LBP) encoding technique was developed to lower the amount of computation and, therefore, reduce the storage requirement of the authentication system. An enhanced voice activity detection (VAD) technique has also been introduced to eliminate non-relevant segments of speech and to improve the overall processing efficiency. Experimental validation of the proposed system indicates that it is effective at providing reliable and efficient multimodal authentication of users on mobile platforms.

Identity verification and digital resource protection are basic roles of technology in today’s world. Traditional methods (passwords, pin codes, physical security tokens) have been staple means of authenticating users, but they are being targeted more than ever via phishing scams, brute force attacks and reused passwords/credentials [4,5]. Therefore, there is a movement toward using biometric verification systems (fingerprints, voice, eye, facial identification). Biometric systems use physical or behavioral traits to verify the identity of users [7,8].Biometrics can provide a multitude of advantages over the traditional authentication methods in the areas of security protection; usability and resistance to theft; but there are a number of variables that could affect the overall performance of a biometric verification system including; environmental conditions; interference caused by other people; and differences in how a trait is collected [6,8].

#### Unimodal vs. multimodal biometric systems

Unimodal biometric systems utilize a single form of identification, which may present limitations, such as variations in how someone presents themselves within a particular group (i.e., intra-class variability), universal usability (i.e., non-universality), and susceptibility to trickery (i.e., spoofing attacks) [8,11]. To mitigate these limitations, multi-modal biometric systems combine different forms of identification into one system, thereby improving reliability, accuracy, and resistance to attack [1,6,18]. Fusion techniques for multimodal systems can operate at various levels, including sensor level, feature level, score level, and decision level, with each technique providing different levels of compromise between performance and computational complexity [[6], [21], [22]]. Many studies show that multibiometric systems (e.g., combinations of face/finger vein [3], ECG/finger vein [1], or iris/face/finger vein [6]) are typically able to perform better in terms of accuracy and resistance to spoofing than unimodal biometric systems [19,20].

#### ECG-based biometric authentication

When it comes to biometric authentication, electrocardiogram (ECG)-based biometrics are proving to be a viable option for individuals who cannot supply traditional biometric information and also meet repeated use cases of continuous authentication. As ECG signals are unique to each person and capable of being liveness-detected, they are not susceptible to spoofing [1,12,41,42]. Research conducted by El-Rahiem et al. [1] and Yeşilkaya & Guest [12] shows that ECGs can be acquired from easily accessible locations (for example, fingertips), or by using wearable technologies, making them an effective source of biometric authentication across a broad range of applications. On the other hand, although ECG signals are quite unique to each person, they are influenced significantly by physiological changes and environmental interference and require robust preprocessing and feature extraction techniques to produce reliable results [42].

#### Machine learning approaches

Traditional methods of machine learning (ML) commonly used in biometric recognition include support vector machine, k-nearest neighbor, and random forest algorithms. Each of these requires thoughtful design and implementation for both feature extraction and pre-processing to convert the raw biometric images into a form of representation usable by the model [14,15,18]. Machine learning-based models are good performers in controlled environments, but these models tend to lack generalization ability when applied to different datasets and conditions of transformation and noise, particularly when images are used for biometric identification, where rotation, size and occlusion can degrade the performance of the model once again [15].

#### Deep learning and hybrid approaches

Since deep learning (DL) does not require that features be hand-crafted, it can learn discriminative features from raw image data; this has allowed it to become more robust against variations within class and against noise [16,23,24,26]. The most widely used architectures for the development of DL for image-based biometric authentication are Convolutional Neural Networks (CNN) [3,13,14]. Advanced hybrid and ensemble approaches further integrate multiple networks or modalities to achieve higher accuracy and security [25,28,31]. Despite these advantages, DL-based systems face challenges such as overfitting, increased computational complexity, susceptibility to adversarial attacks, and the need for large annotated datasets [19,27,29]. Federated learning has recently been explored to address data privacy issues while maintaining high recognition performance in distributed biometric systems [25,36].

To systematically analyze recent advancements in deep learning-based biometric authentication, a comparative study is presented. Table 4 summarizes the methodological aspects, while Table 5 provides performance-based evaluation metrics.”

**Table 4:** General comparison of deep learning-based biometric systems.

Ref. No.	Author (Year)	Modality	DL Technique	Fusion Level	Key Contribution	Advantages	Limitations
[1]	El-Rahiem et al. (2022)	ECG + Finger Vein	Deep CNN Fusion	Feature-level	Fusion of physiological biometrics	High robustness & security	Complex acquisition setup
[3]	Wang et al. (2022)	Face + Finger Vein	CNN	Feature-level	Cross-modal feature fusion	Improved recognition accuracy	High computational cost
[6]	Alay & Al-Baity (2020)	Iris + Face + Finger Vein	Deep Neural Network	Feature-level	Triple-modal biometric system	Very high reliability	System complexity
[10]	Alkanan et al. (2026)	Face + Behavioral	CNN + Behavioral Modeling	Hybrid	IoT-based smart authentication	Real-time & adaptive	Privacy concerns
[12]	Yeşilkaya & Guest (2025)	ECG	Deep Learning (CNN/RNN)	Unimodal	Activity-aware ECG authentication	Continuous authentication	Noise sensitivity
[13]	Boucherit et al. (2022)	Finger Vein	Deep CNN	Feature-level	Deep vein feature extraction	High accuracy	Specialized sensors needed
[14]	Obayya et al. (2020)	Palm Vein	CNN + Bayesian Optimization	Unimodal	Contactless vein recognition	Hygienic & secure	Limited datasets
[23]	Salturk & Kahraman (2024)	Face + Signature	Deep Learning	Feature-level	Behavioral + physical fusion	Robust authentication	Signature variability
[24]	Haider et al. (2023)	Finger Texture + Vein	CNN + Fuzzy Logic	Score-level	Score-level fuzzification	Improved matching accuracy	Increased processing time
[25]	Coelho et al. (2023)	Multimodal	Federated Deep Learning	Feature-level	Privacy-preserving multimodal learning	No centralized data sharing	Communication overhead
[27]	Alharbi & Alshanbari (2023)	Face + Voice	FaceNet + GMM	Score-level	Audio-visual biometric fusion	Robust to modality failure	Noise sensitivity
[28]	Abdullahi et al. (2023)	Fingerprint + Vein	Deep Sequential Network	Feature-level	Sequence-based multimodal learning	High accuracy	Data dependency
[31]	Huang et al. (2023)	Finger (multi-source)	Deep Asymmetric Network	Feature-level	Similarity-based fusion	Enhanced recognition	Model complexity
[35]	Sasikala (2025)	Multimodal	Attention + EfficientNet	Feature-level	Attention-based feature optimization	High efficiency & accuracy	Computational overhead
[36]	Guo et al. (2024)	Multimodal	Federated Learning	Feature-level	Distributed biometric learning	Privacy preservation	Training latency
[37]	Gimba et al. (2025)	Face + Fingerprint	CNN	Feature-level	CNN-based multimodal fusion	High performance	Spoofing risk
[40]	Hussian et al. (2025)	Fingerprint	Deep CNN	Unimodal	Hybrid deep fingerprint model	Strong feature extraction	Limited generalization

**Table 5:** Performance comparison of deep learning-based biometric systems.

Ref. No.	Author (Year)	Modality	Dataset	Accuracy (%)	FAR (%)	FRR (%)
[1]	El-Rahiem et al. (2022)	ECG + Finger Vein	SDUMLA-HMT, ECG-ID	∼98.2	NR	NR
[3]	Wang et al. (2022)	Face + Finger Vein	CASIA-WebFace, SDUMLA-HMT	∼99.1	NR	NR
[6]	Alay & Al-Baity (2020)	Iris + Face + Finger Vein	CASIA, SDUMLA-HMT	∼99.3	NR	NR
[10]	Alkanan et al. (2026)	Face + Behavioral	NR	∼98.5	NR	NR
[12]	Yeşilkaya & Guest (2025)	ECG	ECG-ID, PTB	∼96.8	∼2.1	∼2.5
[13]	Boucherit et al. (2022)	Finger Vein	SDUMLA-HMT, FV-USM	∼98.6	NR	NR
[14]	Obayya et al. (2020)	Palm Vein	PolyU	∼99.0	NR	NR
[23]	Salturk & Kahraman (2024)	Face + Signature	GPDS, CASIA	∼97.5	NR	NR
[24]	Haider et al. (2023)	Finger Texture + Vein	SDUMLA-HMT	∼98.9	∼1.8	∼2.0
[25]	Coelho et al. (2023)	Multimodal	Distributed	∼97.8	NR	NR
[27]	Alharbi & Alshanbari (2023)	Face + Voice	VoxCeleb, LFW	∼98.4	∼1.5	∼1.9
[28]	Abdullahi et al. (2023)	Fingerprint + Vein	SDUMLA-HMT	∼99.2	NR	NR
[31]	Huang et al. (2023)	Finger	PolyU, SDUMLA-HMT	∼98.7	NR	NR
[35]	Sasikala (2025)	Multimodal	CASIA, LFW	∼99.0	NR	NR
[36]	Guo et al. (2024)	Multimodal	Multiple	NR	NR	NR
[37]	Gimba et al. (2025)	Face + Fingerprint	LFW, FVC2004	∼98.8	NR	NR
[40]	Hussian et al. (2025)	Fingerprint	FVC2004, NIST SD4	∼97.9	∼2.3	∼2.6

### Research gaps

Challenges faced by the existing methods are illustrated below.

**Fig. 1:**
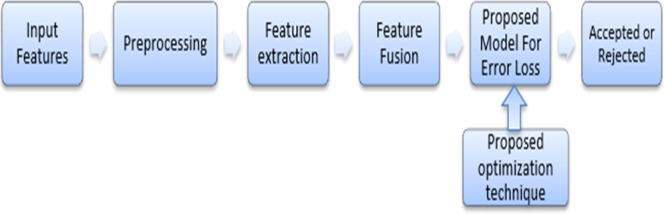
General block diagram.

• The deep CNN model required big data to obtain high performances even though the model had achieved good results on small data. In addition, the model took long time for first time of authentication [1,13].
• In [40], the extended local binary patterns (ELBP) method required more improvements to increase the accuracy, level of authentication, and even more for real time applications.
• When compared to the electrocardiogram (ECG), the group authentication model's performance was worse when the fusion signal was present. Long short-term memory (LSTM) model provided acceptable accurateness and precision. However, a number of legitimate login attempts were denied [41].
• The hybrid multi-phase feature fusion model required optimization to tune the weight parameters of neural network and also required improvements regarding feature extraction, thereby extract handcrafted features for better authentication [42] (Fig. 1)

### Proposed methodology

**Fig. 2:**
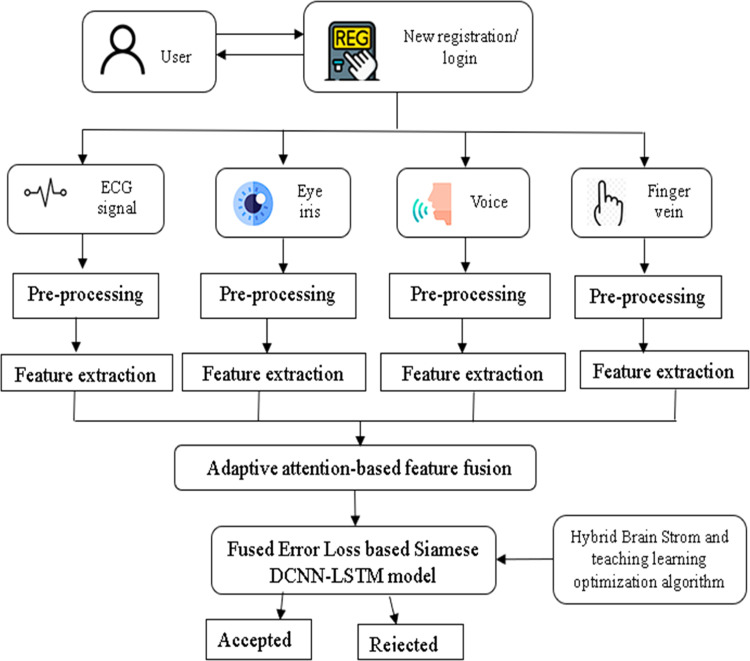
Proposed architecture.

This research aims to design and develop a multi-modal based authentication using Fused error Loss-based Siamese DCNN-LSTM to overcome the limitation of unimodal biometric system, which is more susceptible to different attacks and threats. Initially, for user registration, four biometrics traits such as electrocardiogram (ECG), eye iris, voice, and finger vein from four different dataset such as ECG ID [33], CASIA Iris [30], Finger Vein [31] and AudioMNIST [32] will be taken for this process. These biometric features will be fed into the pre-processing stage, which carries two techniques such as butterworth filter technique for ECG signals as well as voices, and adaptive thresholding technique for iris as well as finger vein traits. Here, the butterworth filter removes artefacts from the signals whereas, the adaptive thresholding technique enhance the image biometric for better authentication. The pre-processed user biometrics will directly fed into feature extraction phase, in which the required features from each biometrics will be extracted for effective authentication. For ECG signal, the features are extracted using techniques such as R-peak detection, PQRST segment feature, Time-amplitude features, and HRV features, which capture only important features for further process. For eye iris, the features are extracted using three different techniques namely Hybrid structural edge features, Modified ternary pattern, and Hybrid residual flow map. Moreover, the important features of voice signals will be extracted using MFCC, Audio content analysis based statistical features, and VGG-16 feature extraction techniques. Then the finger vein trait will be extracted by Hybrid Robert feature, Ridge detection, and Minutiae feature extraction techniques. The outcome of each extracted features will be fused using Adaptive attention-based feature fusion mechanism, which combines the extracted features and provides single vector. This fusion mechanism will increase the learning capability of the proposed model. Now, the fused vectors will be stored in template storage. While login, the stored vector will be fed into the proposed model, Fused Error Loss-Based Siamese Deep Convolutional Neural Network with Long-Short-Term Memory (DCNN-LSTM) for effective authentication. The proposed model has Fused Error Loss, which is the combination of categorical cross entropy and sparse cross entropy techniques that minimize error loss of the proposed model. The parameters of the proposed model will be tuned using newly developed optimization technique, Hybrid Brain Strom and Teaching Learning algorithm, which progress the authentication process. Finally, the model checks the authenticity of the user, if the user is authorized then the model will send accept acknowledgement otherwise rejects it. This proposed model will provide quite high accuracy and less error rates in terms of authentication. A number of metrics, including accuracy, true positive rate (TPR), and false positive rate (FPR), and true negative rate (TNR) will be used to assess the model's performance, and implemented using PYTHON tool. (Fig. 2)

### Datasets used

Public benchmark datasets serve as a widely accepted source for evaluating and comparing biometric authentication methods. Essentially, these datasets provide a set of standard data, which allows researchers to compare performance between different systems and modalities based off of training and test data collected from the same set of subjects within a controlled environment.

#### ECG dataset

The ECG-ID database is the most frequently used collection of electrocardiogram recordings for biometric authentication based on ECG signatures. This database contains recordings made from a number of different individuals under controlled conditions and can also be used to evaluate systems that use signal information from biometric authentication systems.

#### Finger vein dataset

Examples of databases used to create vein recognition systems are the SDUMLA-HMT dataset and other publicly available collections. These datasets are typically composed of NIR images that capture vascular patterns within the human hand or finger. Vascular patterns are often unique to each individual, making them difficult to alter or replicate.

#### Iris dataset

One of the best known and widely used dataset for iris recognition is the CASIA-Iris dataset. It provides a large number of high-quality iris images of human irises that have all been captured under controlled conditions, which are used to benchmark iris-based biometric authentication systems.

#### Audio dataset

The AudioMNIST database provides a collection of recordings of various speakers pronouncing digits. This is commonly used for research on voice based biometric authentication.

All the above-mentioned datasets are publicly available and have been widely utilized in the biometric research community, allowing for reproducibility and a fair and accurate way to compare different methods of biometric authentication.

**Table:** 

Dataset	Modality	No. of Subjects	Data Type	Usage
ECG-ID	ECG	90+	Signal	Authentication
SDUMLA-HMT	Finger vein	100+	NIR images	Recognition
CASIA-Iris	Iris	1000+	Images	Benchmarking
AudioMNIST	Voice	60	Audio	Speaker recognition

## Conclusion

The limitations of a unimodal biometric authentication system are addressed in this paper by proposing a multi-modal based authentication system utilizing fused error loss-based Siamese DCNN-LSTM. The development and deployment of multimodal biometric authentication systems represent a substantial advancement in securing sensitive applications and systems. Incorporating a mix of biometric traits, like ECGs, irises, finger veins, and voices into one biometric authentication platform helps eliminate the limitations of traditional unimodal biometric solutions that fall victim to spoofing attacks, face difficulties with background noise, and cannot differentiate between users due to intra-class variability.

Multimodal biometric authentication systems benefit from the individual biometric modality's strengths working together to provide higher levels of security and usability; reduce false acceptance rates (FAR) and false rejection rates (FRR); and provide flexibility and scalability across a range of settings from personal devices to large-scale applications such as banking, healthcare, and border control. Therefore, multimodal biometric authentication systems represent a viable and efficient method for secure identity verification in today's ever-changing digital world.

## Ethics statements

[Our work did not copied / involved data collection from social media platform].

## CRediT author statement

**Mrs. Shital Kakade:** Conceptualization, Methodology, Writing- Original draft preparation.

**Dr. Umesh Raut:** Supervision, Writing- Reviewing and Editing.

## Declaration of interests

The authors declare the following financial interests/personal relationships which may be considered as potential competing interests:

Mrs. Shital Kakade reports a relationship with Dr. Vishwanath Karad MIT World Peace University, Pune. that includes: non-financial support. If there are other authors, they declare that they have no known competing financial interests or personal relationships that could have appeared to influence the work reported in this paper.
